# Quality Assessment of Reconstructed Cow, Camel and Mare Milk Powders by Near-Infrared Spectroscopy and Chemometrics

**DOI:** 10.3390/molecules29173989

**Published:** 2024-08-23

**Authors:** Mariem Majadi, Annamária Barkó, Adrienn Varga-Tóth, Zhulduz Suleimenova Maukenovna, Dossimova Zhanna Batirkhanovna, Senkebayeva Dilora, Matyas Lukacs, Timea Kaszab, Zsuzsanna Mednyánszky, Zoltan Kovacs

**Affiliations:** 1 Department of Food Measurements and Process Control, Institute of Food Science and Technology, Hungarian University of Agriculture and Life Sciences, 1118 Budapest, Hungary; mariem.majadi@gmail.com (M.M.); lukacs.matyas.krisztian@phd.uni-mate.hu (M.L.); kaszab.timea@uni-mate.hu (T.K.); 2 Department of Livestock Product and Food Preservation Technology, Institute of Food Science and Technology, Hungarian University of Agriculture and Life Sciences, 1118 Budapest, Hungary; annamaribarko@gmail.com (A.B.); toth.adrienn@uni-mate.hu (A.V.-T.); 3 Reference Laboratory of Dairy Products, Kazakh National Agrarian Research University, 050010 Almaty, Kazakhstan; zhulduz.suleimenova@kaznaru.edu.kz (Z.S.M.); janna_90.18@mail.ru (D.Z.B.); 4 Department of Technology and Processing of Livestock Production, S. Seifullin Kazakh Agro-Technical Research University, 010011 Astana, Kazakhstan; dilor1986@mail.ru; 5 Department of Nutrition, Institute of Food Science and Technology, Hungarian University of Agriculture and Life Sciences, 1118 Budapest, Hungary; mednyanszky.zsuzsanna@uni-mate.hu

**Keywords:** amino acid profile, special diet, fingerprinting, technological properties, digitalization, modelling

## Abstract

Milk powders are becoming a major attraction for many industrial applications due to their nutritional and functional properties. Different types of powdered milk, each with their own distinct chemical compositions, can have different functionalities. Consequently, the development of rapid monitoring methods is becoming an urgent task to explore and expand their applicability. Lately, there is growing emphasis on the potential of near-infrared spectroscopy (NIRS) as a rapid technique for the quality assessment of dairy products. In the present work, we explored the potential of NIRS coupled with chemometrics for the prediction of the main functional and chemical properties of three types of milk powders, as well as their important processing parameters. Mare, camel and cow milk powders were prepared at different concentrations (5%, 10% and 12%) and temperatures (25 °C, 40 °C and 65 °C), and then their main physicochemical attributes and NIRS spectra were analyzed. Overall, high accuracy in both recognition and prediction based on type, concentration and temperature was achieved by NIRS-based models, and the quantification of quality attributes (pH, viscosity, dry matter content, fat content, conductivity and individual amino acid content) also resulted in high accuracy in the models. R^2^CV and R^2^pr values ranging from 0.8 to 0.99 and 0.7 to 0.98, respectively, were obtained by using PLSR models. However, SVR models achieved higher R^2^CV and R^2^pr values, ranging from 0.91 to 0.99 and 0.80 to 0.99, respectively.

## 1. Introduction

The dairy sector has seen profound technological changes and significant growth over recent decades [1]. Global milk production is projected to grow at an annual rate of 1.7% and to reach 981 million metric tons by 2028 [2]. The consumption of milk is rapidly increasing, particularly in highly populated and poor regions of the world, where milk is the main source of protein (as meat and other protein-rich foods are rarely available [3]), which can include milk from various animals [4]. This fact, along with the perishable nature of milk, transportation challenges and the lack of access to adequate refrigeration methods, has created an urgent need to enhance processing efficiency in the dairy industry [5,6,7]. Various processing methods have been applied to extend the shelf life of milk, including the manufacturing of milk powders through evaporation and drying techniques. The most common method to produce powdered milk is spray drying. This method is based on the principle of convection, where water is removed from finely dispersed milk particles upon contact with circulating hot dry air, resulting in the almost instantaneous formation of dry product particles with an average diameter of 50 microns [8,9].

Powdered and liquid forms of milk serve similar nutritional purposes but have different characteristics, which make them suitable for various applications. Liquid milk has a shorter shelf life and requires refrigeration, making it more suitable for immediate consumption [10,11]. It is a versatile raw material used in many food formulations due to its creamy texture and flavor. It is commonly used in dairy-based beverages (milkshakes, flavored milks), frozen desserts (ice cream, frozen yogurt) and cereal bars. On the other hand, powdered milk has an extended shelf life and does not require refrigeration, making it ideal for long-term storage and various applications, including industrial use, in specialized diets and in emergency food reserves. It is preferred for applications where precise control of the concentration and moisture content is required due to its ability to be reconstituted [8,9]. Lately, powdered milk has become a major attraction for many industrial applications due to its physical and functional attributes [5]. It is frequently incorporated as an ingredient in the formulation of numerous dairy and processed food products, including ice cream, cultured milk and yogurts, chocolate, confectionery, bakery products, soups and sauces [12,13,14].

Camel and mare milk have gained a lot of attraction in recent years due to their nutritional benefits, their medical attributes and the ability of camels and mares to survive harsh climates. This makes them excellent alternatives to cow milk, particularly in areas where cows struggle to survive [15]. Additionally, camel and mare milk are less prone to causing allergies, making them a perfect substitute for cow milk in certain food applications. Camel milk is homologous to human milk due to its distinctive chemical composition full of bioactive and functional compounds, such as essential amino acids [16]. Hence, there is an increasing interest in investigating the suitability of camel milk as an alternative to cow’s milk-based hypoallergenic infant formulas [17,18]. Currently, no infant formulas based on camel milk are commercially available in the EU market, as further in-depth investigations are required, in addition to the fact that there is limited production of camel milk in the EU [19]. Similarly, mare’s milk has been suggested as a possible alternative to bovine milk in pediatric dietetics [20,21,22]. It has an ideal ratio of caseins and whey proteins, which makes it highly digestible compared to cow’s milk. Additionally, fermented mare’s milk, known as Kumis, is considered a very valuable nutritional drink due to its content of probiotics, such as *Lactobacillus acidophilus* and *Bifidobacterium* [23].

The quality of milk powders and their applicability depend on their chemical, physical and functional attributes, all of which are interdependent [24]. In addition to other physicochemical parameters, the amino acid and fatty acid composition are often used to assess the quality of milk [25], including human breast milk [26,27]. Additionally, the conditions under which milk is reconstructed, such as temperature and concentration, significantly impact these parameters.

Processing conditions, particularly pH and temperature, have a great impact on the above-mentioned parameters. Generally, an increase in temperature leads to a decrease in viscosity [2]. However, when exposed to higher temperatures, the viscosity increases because of protein denaturation. The extent to which an increase in the viscosity occurs was proven to be linked to the applied pH as well, with a significant increase when low pH is coupled to heat treatment and an opposite effect otherwise [28,29]. The insolubility index is another important quality indicator that is highly affected by the impact of pH and temperature conditions on the milk protein behavior. A high insolubility index as a result of milk protein denaturation is considered a serious defect and can lead to the rejection of the milk powder product [24].

Besides its physical and functional properties, powdered milk is valued for its nutritional value and chemical characteristics, mainly titratable acidity, fat, protein content and amino acid profile. The fat content of milk powders can dictate their storage ability and their applicability as well [30]. High fat levels in milk powders can result in them becoming rancid easily; hence, they can reduce their shelf life [13]. Moreover, fat content is an important quality attribute, especially in the manufacturing of chocolate, where milk powder is used as the main ingredient for the preparation of milk crumbs [31]. Titratable acidity is another important parameter that must be monitored, as it is an indicator of the microbiological quality of milk powders. Additionally, the determination of the amino acid profile is crucial, particularly for milk powders destined for the processing of high-value food products such as infant formulas and fortified blended foods for vulnerable groups [32].

The development of rapid monitoring methods has become an urgent task to explore the applicability of quality control of various food products including milk powders and milk reconstructed from milk powder in food formulations, as well as export potential [33,34,35]. Recently, there has been a growing emphasis on the potential of near-infrared spectroscopy (NIRS) for the quality assessment of dairy products. This technique is non-destructive and very environmentally friendly compared to other analytical methods such as gas chromatography, high-performance liquid chromatography, mass spectroscopy and electrophoresis. Additionally, no reagents are required, and no hazardous waste is produced. The NIRS technique has been applied increasingly for food quality evaluation in recent years, and several of these applications are currently in use as routine analyses and in online monitoring systems [36]. NIR is a highly accurate, versatile and multi-analytical technique based on the absorption of near-infrared light by the sample at different wavelengths (800–2500 nm), recording molecular vibration of all molecules containing C-H, N-H or O-H groups [37]. Numerous studies have investigated the applicability of NIRS for quality assessment and classification of different milk powders. Wu et al. [38] investigated the feasibility of the short-wave NIRS technique for the analysis of fat, protein and carbohydrates in milk powder. Additionally, Wang et al. [7] studied the potential of visible–NIRS for the prediction of functional quality attributes of milk powders from different brands, types and sources. In similar studies, Inácio et al. [39] and Chen et al. [40] studied the applicability of NIRS for the classification of milk powders according to their brand and the prediction of their protein content.

While research has been conducted on using NIRS to assess the quality of milk powder, there has been less focus on how NIR can be applied to reconstructed milk, particularly in detecting defects that may occur due to improper reconstruction conditions. Additionally, research on camel and mare milk is considerably scarcer, despite their significant importance in the diets in certain regions and ethnic communities.

Various kinds of milk powders, each possessing unique chemical compositions, can influence food functionality and their ability to better suit specific groups with different ethnic backgrounds. In the present work, we aim to explore the feasibility of NIRS coupled with chemometrics as a novel technique for the rapid characterization of milk powders from different animals (cows, mares and camels). Another objective is to distinguish between different types of milk and various reconstruction conditions using NIR spectra, to evaluate the technique’s effectiveness as a quality control tool.

## 2. Results and Discussion

### 2.1. Characterization of Cow, Camel and Mare Milk Powder Samples and Reconstructed Milk Samples

During storage, water activity needs to be maintained at a certain level to prevent the irreversible crystallization of amorphous lactose, which can lead to the formation of lumps in the milk powder [11]. As reported in Table 1, the tested cow, camel and mare milk powders had average water activity values of 0.24, 0.28 and 0.22, respectively. Similar results were reported in a study conducted by Pugliese et al. [11], where the water activities of milk powders ranged from 0.24 to 0.33.

The insolubility indexes of the analyzed powders ranged from 0.1 to 1.37, where skimmed cow milk powder had the lowest insolubility index and whole mare milk powder had the highest index. Similarly, Pugliese et al. [11] reported insolubility indexes of whole milk powders ranging from 0.1 to 0.8 mL and from <0.1 to 0.1 mL for skimmed milk powders. Additionally, Wang et al. [7] reported insolubility indexes of various types of milk powders ranging from 0.14 mL to 7.54 mL, where skimmed milk powders showed the lowest index as well. According to the International Dairy Federation (IDF) standard, the insolubility index is defined as the measure of the volume of sediment after rehydration, mixing and centrifugation [24]. The behavior of milk proteins has a great impact on the solubility of powders. During rehydration, when proteins denature whether because of changes in temperature or pH conditions, they tend to aggregate and form a sediment at the bottom. Therefore, the insolubility index of the powder increases [12,24]. In the case of whole milk powders, the coagulated proteins with entrapped milk fat globules may float to the surface [5]. A high insolubility index during storage is linked to the Maillard reaction [7].

Bulk density has a great impact on the commercial, economic and functional value of milk powders [31]. Milk powders with high bulk density are preferable because of lower packaging, storage and transportation costs [7,24]. The bulk density varied among the analyzed milk powders from 393.65 to 678.9 kg m^−3^.

Water activity, the insolubility index and bulk density are highly significant quality indicators of milk powders. The ANOVA results showed that the type of dried milk was the most important parameter, presenting significant differences in water activity, bulk density and insolubility index among the tested milk powders. Different processing plants and processing parameters along with external factors such as the type of animal, genetics and breed can dictate the physical abilities of milk powders. Findings by Wang et al. [7] proved that the type of dried milk was the most important parameter that resulted in significant differences between milk powders.

The summary of the two-way ANOVA results for the main quality parameters of the reconstructed milk samples resulting in significant differences based on the concentration or temperature level used to reconstruct the milk is shown in Table 2.

The interaction type* temperature was found to have a significant effect on the conductivity, pH and a* of the milk samples. However, the type of powdered milk was found to have a greater effect on the pH and a* compared to the temperature. Reconstructed camel milk had the lowest pH values (~6.5) compared to the others, which is similar to what was reported by Swelum et al. [41]. Furthermore, the viscosity, titratable acidity, dry matter (%), L* and b* of the milk samples were significantly influenced by the interaction type* concentration. However, the concentration seemed to have more effect on the dry matter content and the viscosity in comparison to the type of powdered milk. The viscosity of the reconstructed milk increased with the increase in the concentration and dry matter content, where reconstructed cow and camel milk had the highest viscosity. The titratable acidity was more affected by the type of powder contrary to the concentration, where reconstructed camel milk had the highest values.

To conclude, most of the significant differences found between cow, mare and camel milk samples reconstructed at different concentrations and temperatures were found to be due to the type of powdered milk.

### 2.2. NIRS Results

#### 2.2.1. Visual Inspection of Spectra and PCA Model

**Figure 1:**
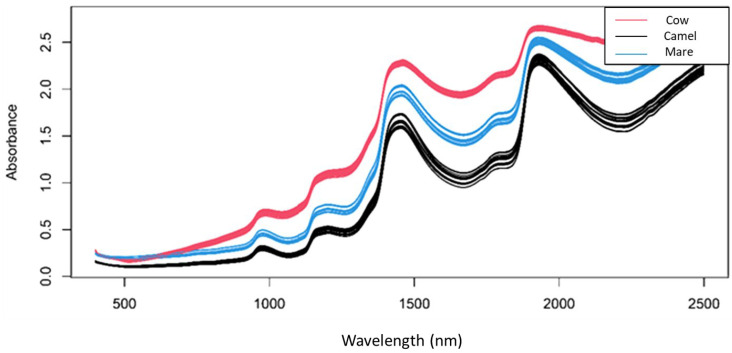
Raw spectra of reconstructed milk samples colored by the type of milk powder (400–2500 nm).

Figure 1 shows the raw near-infrared spectra of cow, camel and mare reconstructed milk samples in the spectral range of 400–2500 nm. Two major peaks were found around 1440 nm and 1950 nm representing the combination of the OH symmetric and asymmetric stretching modes of water and the combination region of the OH stretching and deformation vibrations of water [42]. An apparent difference can be seen between the raw spectra of the reconstructed milk samples, mainly due to their different chemical compositions.

**Figure 2:**
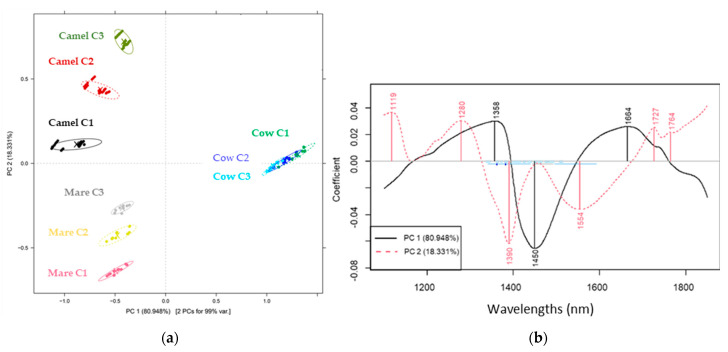
PCA score (**a**) and loading (**b**) plot of reconstructed milk powders from cows, camels and mares colored by their concentration (smoothed and deTrtreated spectra).

Figure 2a shows the PCA score plot of cow, camel and mare milk at three different concentrations. Based on the first two PCs (representing 99.14% of the total variance), a clear separation pattern among the types of milk samples can be observed. This was confirmed in the ANOVA tests as well, where significant differences among the reconstructed milk samples resulted from the type of milk powder. Moreover, the PC2 dimension showed a clear tendency to move apart groups of samples representing different levels of concentration except for the different concentrations of cow milk samples.

The bands representing the highest load in the separation of the groups of cow milk from the other ones are 1358, 1450 and 1664 nm (Figure 2b, PC1), while those contributing to the separation of the groups of camel and mare milk and their different concentrations are 1119, 1280, 1390, 1554, 1727 and 1764 nm (Figure 2b, PC2). The wavelength of 1280 nm is attributed to the first overtone bands of bonded OH-H [43,44]. The band at 1119 nm can be linked with the C-H stretch overtone [43]. Those at 1727 nm and 1764 nm can be assigned to fat and fatty acid absorption due to the first overtone of CH stretching vibrations [45,46]. In addition, 1390 nm was associated with lactose absorption as a result of O-H stretch/O-H bend combination vibration [47]. The band at 1450 nm was attributed to the first overtone of the OH stretching vibrations of water [46]. The band at 1554 nm can be attributed to the first overtone of stretching vibrations of the N-H bonds in amino groups [45,47,48,49], and 1664 nm to the first overtone of aromatic absorption (under C-H) groups [49,50].

The relevant wavelengths and their corresponding functional groups provided key information about the compositional differences between the milk samples based on the type. Therefore, they are considered reliable for accurate model construction.

#### 2.2.2. Discrimination Models Based on Reconstruction Conditions

**Figure 3:**
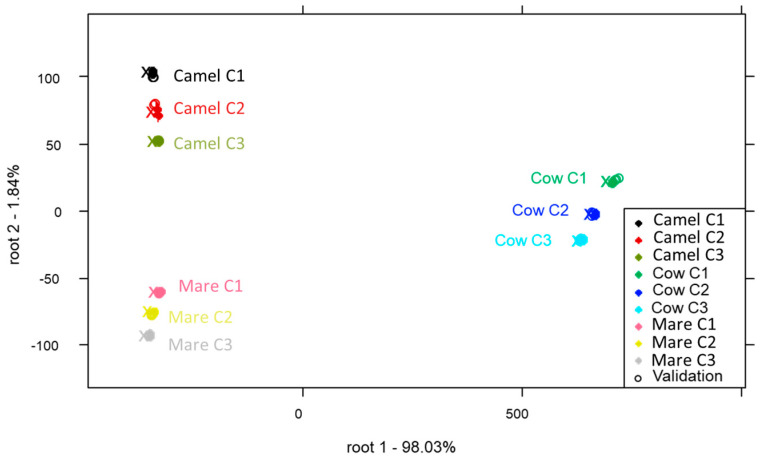
PCA-LDA of reconstructed milk samples at 65 °C (T3) according to the concentration level on MSCtreated spectra. Built with 95% confidence interval ellipses. The center of the ellipses is denoted by x, and training and validation sets are denoted by solid and hollow points, respectively.

Three PCA-LDA models were developed for each temperature level (25 °C, 40 °C and 65 °C) used to reconstruct the milk to discriminate between the type of milk and the applied concentration. Regardless of the temperature, all classification models demonstrated excellent discrimination between sample groups representing different types of milk and concentrations, achieving an average recognition and prediction accuracy of 100%. For instance, the PCA-LDA score plot developed for the classification of the type of milk and the applied concentrations in the case of samples prepared at 65 °C is presented in Figure 3. Chen et al. [40] reported that milk powders from different brands analyzed with NIRS were 100% correctly classified by using the partial least squares discriminant analysis (PLS-DA) method. Additionally, a good separation between milk powders according to their source (bovine, goat, soy-based) was achieved using NIRS and PCA in a study by Wang et al. [7].

**Figure 4:**
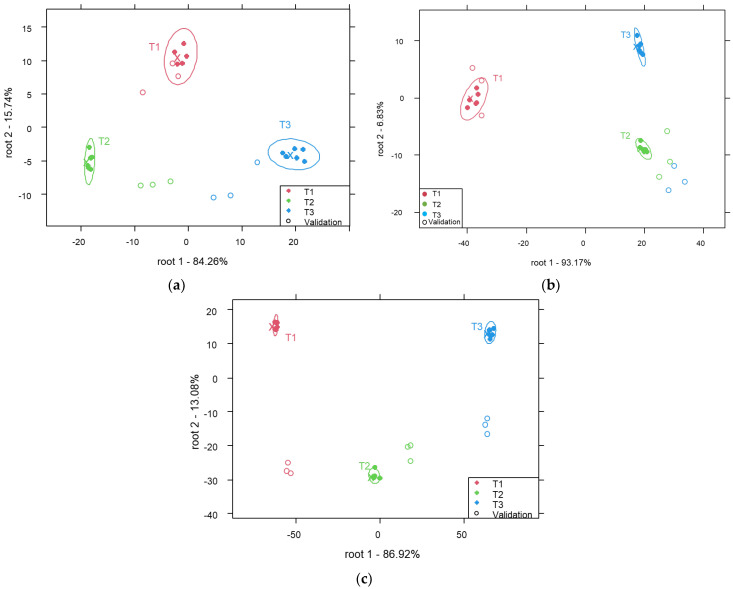
PCA-LDA score plot for the classification of reconstructed cow (**a**), camel (**b**) and mare (**c**) milk samples at a concentration of 5% according to the temperature of preparation (smoothed spectra (**a**), smoothed and SNV-treated spectra (**b**) and smoothed and MSCtreated spectra (**c**)).

Figure 4 shows the PCA-LDA models developed for the classification of reconstructed milk samples at a concentration of 5% according to their temperature of preparation. A classification model was developed for each type of milk. In general, the models allowed for good discrimination between groups of samples representing different temperatures of preparation. For instance, the following was observed:

The classification model for the discrimination between cow milk samples (Figure 4a) showed average recognition and prediction accuracies of 100% and 81.5%, respectively. After cross-validation, 33.3% and 22.2% of samples belonging to T2 were misclassified as sample representing temperature levels T1 and T3, respectively.

Average recognition and prediction abilities of 100% were found for the classification of the camel milk samples (Figure 4b). All samples were correctly classified (100%) based on the temperature.

The mare milk model (Figure 4c) presented average recognition and prediction accuracies of 100% and 66.7%, respectively. After cross-validation, 33.3% of the samples prepared at T2 were incorrectly predicted as samples prepared at T3.

Overall, the classification models demonstrated high accuracies in discriminating the samples based on their type, concentration and temperature. However, the discrimination between the types and concentrations was more accurate compared to that for the temperatures of reconstruction.

#### 2.2.3. PLSR and SVR Models

The optimal pretreatment and main model parameters of the SVR and PLSR models developed for the prediction of the main quality parameters of the reconstructed milk samples are summarized in Table 3, Table 4, Table 5 and Table 6. The optimum spectral pretreatment and the best model were determined according to the minimum root mean square error of cross-validation (RMSECV) and prediction (RMSEP) and the highest coefficients of determination of cross-validation (R^2^CV) and prediction (R^2^pr). The optimum number of latent variables was determined by locating a “knee” drop in the scree plot of RMSECV. All SVR models were built by using the first two principal components (PC1 and PC2) as input variables, a cost parameter value of 10 and a radial kernel function.

All the measured physicochemical parameters (Table 3 and Table 4) could be predicted with high accuracies and low predictive errors. For PLSR, R^2^CV and R^2^pr ranged from 0.8058 to 0.9936 and 0.7081 to 0.9884, respectively. SVR models could reach notably better results overall, with R2CV and R2pr values of 0.9144 to 0.9980 and 0.8052 to 0.9783, respectively. The PLS model for the prediction of fat content developed using sgol@2−21−0 pretreatment and a latent variable of 4 performed the best, with R^2^CV and R^2^pr values of 0.8058 and 0.7081 and RMSECV and RMSEP values of 0.4535% and 0.5153%, respectively. This model is not considered the best in comparison to the other measured parameters. This could be explained by the fact that the freezing and thawing of the milk samples influenced their water structure and homogenization [51]. The radial kernel-based SVR model could reach respective R2CV and R2pr values of 0.9347 and 0.8052 and RMSECV and RMSEP values of 0.2535% and 0.4175% to predict fat content. Although these performance metrics are visibly better, there are a number of points to be considered when interpreting them. In the case of SV-based modelling tools, the type of cross-validation applied has some limitations, wherein removing too many samples might alter the support vectors, while removing too few (non-SV samples) might not affect the model at all [52]. Applying a leave-three-consecutives-out CV (as used for PLSR) resulted in the latter for most models built, where the calibration and cross-validation results were almost identical, producing the possibility of over-optimistic predictions. This was further verified by the sizeable gap between the CV and independently predicted results, where a noticeable standard deviation between the use of the individual prediction sets was also observed (±0.1024 and ±0.1383% for R2pr and RMSEP, respectively). For the sole purpose of having identical setups for the two modelling tools, CV was left unchanged.

In a study conducted by Wu et al. [38], the PLS model for fat content prediction in infant milk powder by using short-wave NIRS at the 800–1050 nm region presented a higher R^2^C of 0.945. On the other hand, Aernouts et al. [45] also reported better results in a study where the fat content of raw cow milk was predicted with an R^2^CV equal to 0.996 by using NIRS in reflectance mode with a spectral range of 1000–1700 nm.

The prediction of the amino acid profile of milk powders plays an important role in determining their potential usage in the processing of high-value products including infant formulas and food products destined for malnourished or vulnerable groups in the population [32].

Regarding individual amino acid content, the resulting PLSR models had high R^2^CV and R^2^pr values ranging from 0.9202 to 0.9837 and 0.789 to 0.9202, respectively. The amino acids lysine, leucine and phenylalanine are essential and crucial components found in milk. They are among the primary essential amino acids and are considered highly significant in terms of nutritional value. The accurate prediction of these amino acids demonstrated the highest R^2^CV and R^2^pr values. Glutamic acid, the most abundant amino acid in milk, was estimated the best with R^2^CV and R^2^pr values of 0.9722 and 0.9456, respectively. In comparison, McDermott et al. [53] developed PLS models for the prediction of free amino acid content in bovine milk using NIRS. The obtained models had weak to moderate prediction accuracies with coefficients of correlation in cross-validation (r_c_) ranging from 0.51 to 0.75. Applying SVR as a calibration tool to predict amino acid content produced better performance metrics compared to PLSR in almost all cases. Most notably, the predictive accuracies in the case of the prominent amino acids leucine, phenylalanine and glutamic acid could reach values between 0.9539 and 0.9721 with average prediction errors below 0.05%. The higher predictive accuracies of SVR models could be attributed to the algorithm’s capability to capture severe intrinsic nonlinearities that are often present in complex, multicomponent natural systems, such as milk powders [54]. PLSR, on the other hand, assumes a linear correlation between spectra and property, an assumption that is rarely fully satisfied when observing biological samples with strong intermolecular and intramolecular interactions [55]. A better performance for SV-based (nonlinear) modelling compared to linear PLS has been reported by multiple studies when observing milk samples with NIRS [52,54,56].

**Figure 5:**
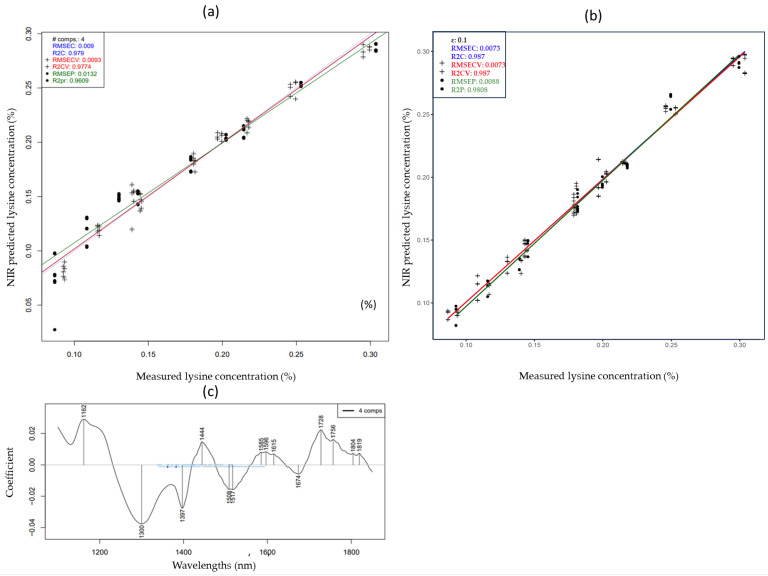
PLSR Y fit (**a**), SVR Y fit (**b**) and PLSR regression vector (**c**) for the prediction of lysine concentrations of cow, mare and camel reconstructed milk samples at different concentrations and temperatures (smoothed and MSCtreated spectra).

The lysine prediction model exhibited the highest accuracies among all measured amino acids. The corresponding PLSR and SVR models are shown in Figure 5. The wavelengths that contributed the most to the prediction of lysine using PLSR are shown in Figure 5c. Peaks located between 1330 and 1600 nm were attributed to the first overtone of O-H and N-H in amino groups [50].

## 3. Materials and Methods

### 3.1. Analyzed Milk Powders

Three types of commercial milk powder purchased from the market were used in this study. Skimmed cow milk powder from Budapest, Hungary, by a brand called Tutti, and whole camel and mare milk powders from brands called Saumal and Sydyk obtained in Almaty, Kazakhstan, were investigated.

### 3.2. Preparation of Reconstructed Milk Samples

Considering the limited number of milk powder samples available in the market, particularly in the case of mare and camel milk, we attempted to incorporate different variations during the reconstruction of the milk powder to allow characteristics such as concentration and temperature to have more representativity in our models.

Milk powders from cow, camel or mare milks were mixed with Milli-Q water to create samples at three different concentrations (C1 = 5%, C2 = 10%, C3 = 12.5%). These mixtures were then dissolved at three different temperatures (T1 = 25 °C, T2 = 40 °C or T3 = 65 °C), with three replicates performed for each combination of concentration and temperature.

All prepared samples were mixed by using a vortex mixer for 6 min to ensure the homogeneity of the samples. This preparation resulted in a total of 81 samples (3 milk powders × 3 concentrations × 3 temperatures × 3 repeats). After cooling, the prepared milk samples were immediately refrigerated and stored at 4 °C until further analysis.

### 3.3. Characterization of the Milk Powder Samples

The following main quality parameters of the milk powder samples were determined in triplicate.

#### 3.3.1. Water Activity

The water activity (aw) of the milk powders was measured at 25 °C using a Novasina LabMaster-aw neo device (Novasina AG, Switzerland) with internal temperature control (0–60 °C). The sample cup was filled with 1.5 ± 0.5 g of milk powder and placed in the measurement chamber, and the cover was tightly closed. Once stability had been reached, the water activity of the sample was read at 25 °C.

#### 3.3.2. Loose Bulk Density

The density of milk powders was measured as loose bulk density. Loose density was measured by weighing a 100 mL calibrated cylinder filled with dried milk that was carefully leveled, without compacting, until reaching an established level, and expressed with the following expression:

loose density (kg m^−3^) = powder weight (kg)/powder volume (m^3^).

#### 3.3.3. Insolubility Index

The insolubility index of the milk powders was analyzed as described by Pugliese et al. [11]: A total of 10 g of dried milk powder was properly mixed with 100 mL of water at 25 °C for approximately 5 min. The obtained suspension rested for 5–15 min, then it was stirred with a spatula. A volume of 50 mL was centrifuged for 5 min at 5× *g*. After the removal of the supernatant, the tube was filled up again with water. The tube was centrifuged in the same manner. The volume of the sediment was then noted down. Additionally, the sediment was dried in an oven at 70 °C until it reached a constant weight. The insolubility index is expressed both as the volume of wet residue (mL), according to the IDF method, and as the weight of the sediment after drying (mg).

#### 3.3.4. Amino Acid Profile

Totals of 80–120 mg of milk powder samples were hydrolyzed in a closed hydrolyzing vessel (KUTESZ, Budapest, Hungary) at 110 °C for 24 h in a blocked thermostat with 10 mL 6 M HCl under a nitrogen atmosphere (FALC Instruments, Treviglio, Italy). Following neutralization (10 mL of 4 M NaOH), samples were filtered twice, first through a standard paper filter and then through a 0.22 µm membrane filter (Nalgene, Rochester, NY, USA). An Automatic Amino Acid Analyzer AAA400 (Ingos Ltd., Prague, Czech Republic) equipped with an Ionex Ostion LCP5020 cation-exchange column (220 × 37 mm) was used for the analysis. After post-column derivatization with a ninhydrin reagent, colorimetric detections were achieved at 570 nm and 440 nm.

The assay was carried out in a strongly acidic medium, with a series of eluents of gradually weakening acidity, with step gradient elution (buffer 1: 0.18 M Li citrate, pH 2.80; buffer 2: 0.20 M Li citrate, pH 3.05; buffer 3: 0.36 M Li citrate, pH 3.35; buffer 4: 0.33 M Li citrate, pH 4.05; buffer 5: 1.20 M Li citrate, pH 4.65). Chromatograms were evaluated using the CHROMuLAN082 program, by comparison with standard amino acid mixtures.

### 3.4. Characterization of Reconstructed Milk Samples

The following main quality parameters of the reconstructed milk powder samples were determined in triplicate.

#### 3.4.1. Dry Matter Content

Dry matter content is expressed as a percentage by mass of matter remaining after completion of the specified drying process. The measurement was determined by drying around 2 g of milk samples in a classic drying oven at 105 °C for constant mass.

#### 3.4.2. pH and Conductivity

The pH and the electrical conductivity of the samples were measured with a dual pH/conductivity meter (Mettler Toledo SevenMulti, Columbus, OH, USA). Before the measurement, the instrument was calibrated at a given temperature, currently established at 25 °C.

#### 3.4.3. Acidity According to Soxhlet–Henkel (Titratable Acidity)

Titratable acidity was recorded as the Soxhlet–Henkel degree (SH°). The measurement was performed by titration with 0.1 N NaOH solution with a phenolphthalein indicator. The following formula was used to obtain the acidity of the milk in terms of Soxhlet–Henkel (°SH) acidity:

°SH = Added NaOH mL × NaOH factor × 2.

#### 3.4.4. Viscosity

The flow curves of the reconstituted milk samples were measured at a temperature of 25 ± 0.2 °C with an MCR302 modular compact rheometer (Anton Paar, Austria) in coaxial cylindrical geometry (CC27) using Rheo Compass software (version 3.63). In the first stage of the measurement, the shear rate increased from 0.1 s^−1^ to 1000 s^−1^ with a linear scale, and 30 points were recorded with decreasing acquisition according to the logarithmic scale between 10 s and 2 s. In the second stage, the viscosity values were recorded every three seconds and resulted in 30 points at a shear rate of 1000 s^−1^. The apparent viscosity at a 750 s^−1^ shear rate in the increasing stage and the average value of the dynamic viscosity at a constant maximum shear rate were determined. 

#### 3.4.5. Fat Content

The determination of total fat content (*v*/*v*%) was carried out from the resolved samples using the Gerber (acido-butyrometric) method. The standards ISO 2446 [57] and IDF 105 [58] were used for this analysis. The method was carried out as described by Trout and Lucas [59] and Esen and Güzeler [60].

#### 3.4.6. Color Properties

The color properties were measured using a ColorLite sph 850 spectrophotometer (ColorLite GmbH., Katlenburg-Lindau, Germany) over wavelengths ranging from 400 to 700 nm, with a D65 light source and a 2° observer angle. The results were expressed as CIE (Commission Internationale de la Éclargie) L*, a*, b* color parameters.

#### 3.4.7. NIRS Analysis

The NIR spectra of the reconstructed milk samples were collected in transflectance mode with an XDS Rapid Content Analyzer (Metrohm, Denmark) in the wavelength range of 400–2500 nm. The samples were scanned in random order and about 1.5 mL of the reconstructed milk sample was poured in a transflection vessel and covered with a gold reflector plate, resulting in 0.5 mm layer thickness. Three consecutive scans were performed on each sample at a spectral resolution of 0.5 nm. Spectral acquisition was carried out at room temperature (25 °C).

### 3.5. Data Analysis

Descriptive statistics and two-way analysis of variance (ANOVA) were performed on the generated data using the software IBM SPSS27 (Armonk, NY, USA, 2020) as a statistical evaluation tool. Two-way ANOVA tests were conducted to assess the differences between groups of samples belonging to different types of milk powder, concentrations and temperatures used to reconstruct the milk. The normality of residuals of all dependent variables was checked by the Kolmogorov–Smirnov (KS) test. In addition, the homogeneity of variances was checked for all dependent variables according to Levine’s test. In the case of significant two-way ANOVA results (*p* < 0.05), a post hoc test was run using Tukey’s (in the case of homogeneity of variance) or the Games–Howell test (in the case of non-homogeneity of variance) to evaluate differences between groups.

Chemometric analysis of the NIRS data was performed with R-project software (version 4.3.1) [61] using the package aquap2 [62] with principal component analysis (PCA), linear discriminant analysis (LDA), partial least square regression (PLSR) and support vector regression (SVR) methods.

The chemometric analysis in this study was performed in the range of 1100–1850 nm since the most relevant wavelengths for building the classification and regression models were mainly concentrated in the long spectral wavelength region. However, absorbance higher than 2 is beyond the linear response region of the detector [63]. Therefore, wavelengths beyond 1850 nm were not considered in the chemometric analysis.

A preliminary examination of the spectral datasets for unusual or outlying observations was performed by principal component analysis-based linear discriminant analysis (PCA-LDA). PCA-LDA was used to build classification models for the discrimination of the type of reconstructed milk, the preparation temperature and the applied concentration. In addition, three classification models were developed for each temperature level used to reconstruct the milk to discriminate between the type of milk and the applied concentration. For each type of milk, three classification models were developed for each applied concentration to discriminate between the temperature levels used to reconstruct the milk. The PLSR technique was used to build regression models for the quality attributes of the reconstructed milk samples.

The validation of the PCA-LDA models was performed by using the leave-one-repeat-out cross-validation method, whereby data representing one repeat were excluded from the training set and used as a validation set whilst the other two-thirds of the data (two repeats) were used for model building. This procedure was repeated three times, using a different repeat (1/3 of data) in the validation set each time. PLSR models were validated by using leave-one-replicate-out (three consecutive scans together) cross-validation. To prevent model overfitting when dealing with a limited number of samples, an appropriate data division method for creating training and test sets was also employed (test-set prediction) by using two-thirds of the data for training and one-third for predicting. In this study, we attempted to fairly divide the data by considering various factors such as concentration and temperature of reconstruction, replicates and consecutive scans. We aimed to ensure the fairness by adhering to these principles: (1) all consecutive scans of the same sample were placed in either training or test sets, (2) maintaining an equal ratio between training and test sets for samples within each temperature and concentration category. In the case of SVR, the same validation procedure was applied for comparative reasons. Dimension reduction was achieved via PCA by setting the threshold to 95% of the total variance; selected PCs were applied as input variables for SVR modelling [52,64]. Hyperparameter selection was achieved by tuning the error weight (C: 0.1–10) and maximal error value (ε: 0.01–0.5) parameters and by testing with different kernel functions (linear, polynomial and radial). The cost function was used to simultaneously minimize the coefficients and prediction errors to obtain the best performing model for each parameter [55].

Spectral pretreatments were applied to the raw spectra before developing any models to reduce the noise (smoothing techniques) and increase the signal from the chemical information (derivation, normalization, differentiation, etc.). The applied pretreatments included multiplicative scatter correction (MSC), the standard normal variate (SNV), the Savitzky–Golay second derivative with a 21-point window gap (Sgolay 2-21-0), de-trending (deTr) and a combination of these as well.

## 4. Conclusions

Milk powders offer many functional, nutritional and economic advantages for various industrial applications such as food formulations, among others. Having a deep understanding and investigating the general properties of milk powders are primordial tasks for the above-mentioned applications. Although it is traditionally analyzed in a powder state, our research aims to study the feasibility of NIRS combined with chemometrics for rapid determination of general quality attributes of reconstructed milk from various sources (cow, camel and mare). Differences between samples according to the type of milk powder, concentration (5%, 10% and 12.5%) and temperature (25 °C, 40 °C and 65 °C) were investigated using ANOVA tests and linear discriminant analysis. ANOVA tests revealed that the type of milk powder used for the reconstruction of the milk samples contributed the most to the significant differences found between the samples. This was confirmed in the PCA-LDA models as well, where milk samples were 100% correctly classified according to their type.

Relevant wavelengths and their corresponding functional groups provided key information about the compositional differences between the milk samples based on the type. Therefore, they are considered reliable for accurate model construction. The classification models, which are based on type, concentration and temperature, demonstrated high accuracies in both recognition and prediction. Additionally, the quantitative models could accurately predict the pH, viscosity, dry matter, fat content, conductivity and individual amino acid content of the reconstructed milk. However, the SVR models showed higher accuracies compared to the PLSR models.

Our research shows that NIRS is a reliable and versatile analytical method for both qualitative and quantitative analysis of reconstructed milk from various animal sources, including cow, camel and mare milk. Through qualitative analysis, NIRS allows for the identification of milk types and the detection of adulteration or contamination. By analyzing the spectral fingerprints of the milk powders, NIRS can differentiate between cow, camel and mare milk, as well as identify any deviations from standard profiles that may indicate quality defects or the presence of unwanted additives. Quantitatively, it offers an accurate determination of key nutritional and compositional parameters. The technique’s ability to generate accurate quantitative and qualitative data without the need for extensive sample preparation or chemical reagents positions it as a promising method for routine quality checks and product standardization in industrial settings. Additionally, NIRS has high potential in various applications in research and development. For instance, it can be used to monitor the effects of different processing conditions on milk powder composition, optimize formulations for specific nutritional requirements and support the development of new dairy products. The technique’s rapidity and user-friendliness also enable high-throughput screening, making it suitable for large-scale studies or production settings.

While our current models provide reliable results, the representativity and predictive accuracy can be significantly enhanced by broadening the dataset. Incorporating milk powders from a wider array of processing conditions, such as variations in drying techniques, thermal treatments and storage conditions, will help capture the full spectrum of variability in milk powder characteristics. Additionally, including samples from different brands and geographic locations will ensure that the models are robust and generalizable across different market segments and regions. To further improve the reliability and applicability of NIRS in milk powder analysis, future research should focus on expanding the calibration and validation datasets, exploring additional advanced nonlinear chemometric techniques for model development and integrating NIRS with other complementary analytical methods. Such research will contribute to the establishment of NIRS as a standard tool in the dairy industry, capable of meeting the growing demands for quality, safety and innovation in dairy products. To conclude, NIRS has high potential in the endeavor of the development of food production using modern digitalization tools.

## Figures and Tables

**Table 1:** Mean ± SD of water activity, insolubility index and bulk density for each type of reconstructed milk powder (cow, camel and mare).

Measured Parameters	Cow Milk Powder	Camel Milk Powder	Mare Milk Powder
Water activity	0.24 ± 0.006 ^a^	0.28 ± 0.002 ^b^	0.22 ± 0.08 ^c^
Insolubility index (mL)	0.1 ± 0.005 ^a^	0.75 ± 0.03 ^b^	1.37 ± 0.02 ^c^
Bulk density (kg m^3^)	678.9 ± 14.1 ^a^	393.65 ± 34.75 ^b^	549.76 ± 16.21 ^c^

Different letters (^a–c^, read vertically) assigned for significantly different groups (*p* < 0.05).

**Table 2:** Mean ± SD of the apparent viscosity, conductivity, pH, titratable acidity, dry matter and color parameters (L*, a*, b*) of cow, camel and mare milk samples reconstructed from milk powders at different concentrations (C1 (5%), C2 (10%), C3 (12.5%)) and temperature levels (T1 (25 °C), T2 (40 °C), T3 (6 5°C)).

Reconstructed Milk Powder				
Measured Parameters	Significant Factors	Cow Milk	Camel Milk	Mare Milk
Apparent viscosity (mPa s^−1^)	C1	3.88 ± 0.05 ^Aa^	3.96 ± 0.05 ^Ba^	3.63 ± 0.06 ^Ca^
	C2	4.20 ± 0.06 ^Ab^	4.25 ± 0.08 ^Ab^	3.82 ± 0.05 ^Bb^
	C3	4.53 ± 0.07 ^Ac^	4.64 ± 0.13 ^Ac^	4.00 ± 0.04 ^Bc^
Conductivity (mS cm^−1^)	T1	6.41 ± 0.03 ^Aa^	5.17 ± 0.12 ^Ba^	2.25 ± 0.02 ^Ca^
	T2	7.36 ± 0.11 ^Ab^	5.93 ± 0.04 ^Bb^	2.55 ± 0.09 ^Cb^
	T3	8.32 ± 0.06 ^Ac^	6.45 ± 0.06 ^Bc^	2.71 ± 0.03 ^Cc^
pH	T1	6.73 ± 0.06 ^Aa^	6.57 ± 0.05 ^Ba^	7.19 ± 0.04 ^Ca^
	T2	6.71 ± 0.06 ^Aa^	6.53 ± 0.08 ^Ba^	7.12 ± 0.07 ^Cb^
	T3	6.72 ± 0.05 ^Aa^	6.51 ± 0.06 ^Ba^	7.23 ± 0.04 ^Ca^
Titratable acidity (°SH)	C1	6.36 ± 0.7 ^Aa^	6.32 ± 0.44 ^Aa^	2.00 ± 0.15 ^Ca^
	C2	6.35 ± 0.29 ^Aa^	6.90 ± 0.22 ^Bb^	2.38 ± 0.19 ^Cb^
	C3	6.95 ± 0.12 ^Ab^	7.32 ± 0.23 ^Bc^	2.56 ± 0.21 ^Cb^
Dry matter (%)	C1	9.08 ± 0.31 ^Aa^	8.75 ± 0.30 ^Ba^	8.93 ± 0.14 ^ABa^
	C2	11.18 ± 0.23 ^Ab^	10.80 ± 0.14 ^Bb^	10.82 ± 0.16 ^Bb^
	C3	13.5 ± 0.46 ^Ac^	12.57 ± 0.13 ^Bc^	12.77 ± 0.68 ^Bc^
L*	C1	68.56 ± 0.72 ^Aa^	61.01 ± 0.85 ^Ba^	57.51 ± 0.77 ^Ca^
	C2	69.90 ± 0.63 ^Ab^	62.69 ± 0.50 ^Bb^	59.97 ± 0.68 ^Cb^
	C3	70.75 ± 0.47 ^Ac^	63.84 ± 0.60 ^Bc^	61.67 ± 0.47 ^Cc^
a*	T1	−0.74 ± 0.05 ^Aa^	−3.01 ± 0.06 ^Ba^	−0.92 ± 0.04 ^Ca^
	T2	−0.77 ± 0.07 ^Aa^	−3.20 ± 0.03 ^Bb^	−1.00 ± 0.05 ^Cb^
	T3	−0.74 ± 0.05 ^Aa^	−3.33 ± 0.07 ^Bc^	−1.02 ± 0.04 ^Cb^
b*	C1	−2.57 ± 0.13 ^Aa^	−5.73 ± 0.30 ^Ba^	−5.18 ± 0.12 ^Ca^
	C2	−1.91 ± 0.12 ^Ab^	−4.69 ± 0.23 ^Bb^	−4.45 ± 0.12 ^Cb^
	C3	−1.36 ± 0.10 ^Ac^	−3.97 ± 0.26 ^Bc^	−3.94 ± 0.08 ^Bc^

Different letters (^a–c^ or ^A–C^) were assigned for significantly different groups. Lower case letters present results for one type of powdered milk (read vertically). Upper case letters present results for the three types of powdered milk (read horizontally). Only parameters which were significantly affected are listed.

**Table 3:** Summary table of the PLSR models for the prediction of the viscosity (mPa s^−1^), conductivity (mS cm^−1^), pH, titratable acidity (°SH), dry matter (%), fat content (%) and color measurement (l*, a* and b*) of cow, camel and mare reconstructed milk samples.

Measured Parameters	LV	Pretreatment	R^2^C	RMSEC	R^2^CV	RMSECV	R^2^pr	RMSEP
Viscosity	5	sgol@2−21−0	0.9198	0.0914	0.9121	0.0956	0.9004	0.0996
Conductivity	5	sgol@2−21−0	0.9861	0.248	0.9848	0.2592	0.9819	0.2836
pH	5	sgol@2−21−0	0.9801	0.0398	0.9785	0.0413	0.9652	0.0528
Titratable acidity	4	sgol@2−21−0	0.9543	0.4314	0.9506	0.4484	0.9299	0.5816
Dry matter	4	sgol@2−21−0	0.9174	0.4814	0.9122	0.4964	0.8662	0.6021
Fat content	4	sgol@2−21−0	0.9267	0.2797	0.8058	0.4535	0.7081	0.5153
L*	4	sgol@2−21−0	0.9747	0.7068	0.9726	0.7353	0.9558	0.9421
a*	4	sgol@2−21−0	0.9938	0.0862	0.9936	0.0862	0.9884	0.1203
b*	4	sgol@2−21−0	0.9795	0.2042	0.9778	0.2124	0.9701	0.2438

**Table 4:** Summary table of the SVR models for the prediction of the apparent viscosity (mPa s^−1^), conductivity (mS cm^−1^), pH, titratable acidity (°SH), dry matter (%), fat content (%) and color measurement (l*, a* and b*) of cow, camel and mare reconstructed milk samples. Number of PCs = 2; C parameter = 10; radial kernel in all cases.

Measured Parameters	ε	Pretreatment	R2C	RMSEC	R2CV	RMSECV	R2pr	RMSEP
Viscosity	0.01	sgol@2−21−0	0.9604	0.0640	0.9604	0.0640	0.9404	0.0784
Conductivity	0.01	sgol@2−21−0	0.9980	0.0945	0.9980	0.0945	0.9974	0.1063
pH	0.1	sgol@2−21−0	0.9821	0.0380	0.9821	0.0380	0.9783	0.0417
Titratable acidity	0.01	sgol@2−21−0	0.9774	0.3108	0.9774	0.3107	0.9638	0.3877
Dry matter	0.1	sgol@2−21−0	0.9144	0.4874	0.9144	0.4874	0.8976	0.5293
Fat content	0.1	sgol@2−21−0	0.9348	0.2536	0.9347	0.2535	0.8052	0.4175
L*	0.1	sgol@2−21−0	0.9878	0.4939	0.9877	0.4938	0.9837	0.5631
a*	0.01	sgol@2−21−0	0.9979	0.0497	0.9979	0.0497	0.9957	0.0708
b*	0.01	sgol@2−21−0	0.9966	0.0825	0.9966	0.0825	0.9952	0.0961

**Table 5:** Summary table of the PLSR models for the prediction of the amino acid content (%) of cow, camel and mare reconstructed milk samples.

Measured Amino Acids	LV	Pretreatment	R^2^C	RMSEC	R^2^CV	RMSECV	R^2^pr	RMSEP
Alanine	4	sgol@2−21−0, msc	0.9757	0.0042	0.9739	0.0044	0.8783	0.011
Arginine	4	sgol@2−21−0, msc	0.9849	0.0049	0.9837	0.005	0.9399	0.0095
Asparagine	4	sgol@2−21−0, msc	0.9837	0.0082	0.9825	0.0086	0.9518	0.0158
Cysteine	4	sgol@2−21−0	0.9478	0.005	0.9434	0.0052	0.789	0.0089
Glutamic acid	4	sgol@2−21−0, msc	0.9742	0.0304	0.9722	0.0315	0.9456	0.0519
Glycine	4	sgol@2−21−0, msc	0.9272	0.0064	0.9204	0.0067	0.9165	0.006
Histidine	4	sgol@2−21−0	0.9463	0.0041	0.939	0.0044	0.931	0.0053
Isoleucine	4	sgol@2−21−0	0.9442	0.0038	0.937	0.004	0.9061	0.0056
Leucine	4	sgol@2−21−0, msc	0.9804	0.0104	0.9789	0.0107	0.9526	0.0178
Lysine	4	sgol@2−21−0, msc	0.979	0.009	0.9774	0.0093	0.9609	0.0132
Methionine	4	sgol@2−21−0	0.9303	0.0047	0.9202	0.005	0.8863	0.0075
Phenylalanine	4	sgol@2−21−0	0.9684	0.0063	0.9651	0.0066	0.9251	0.0105
Proline	4	sgol@2−21−0, msc	0.9656	0.0119	0.9631	0.0124	0.9348	0.0175
Serine	4	sgol@2−21−0, msc	0.9822	0.006	0.9809	0.0062	0.9368	0.0129
Threonine	4	sgol@2−21−0	0.9419	0.007	0.934	0.0075	0.9015	0.0095
Tyrosine	4	sgol@2−21−0	0.9603	0.0083	0.9566	0.0087	0.9119	0.0118
Valine	4	sgol@2−21−0, msc	0.9759	0.005	0.9738	0.0053	0.8355	0.0139

**Table 6:** Summary table of the SVR models for the prediction of the amino acid content (%) of cow, camel and mare reconstructed milk samples. Number of PCs = 2; C parameter = 10; radial kernel in all cases.

Measured Amino Acids	ε	Pretreatment	R2C	RMSEC	R2CV	RMSECV	R2pr	RMSEP
Alanine	0.1	sgol@2−21−0, msc	0.9712	0.0047	0.9711	0.0047	0.9305	0.0070
Arginine	0.1	sgol@2−21−0, msc	0.9835	0.0050	0.9835	0.0050	0.9634	0.0074
Asparagine	0.1	sgol@2−21−0, msc	0.9853	0.0081	0.9853	0.0081	0.9728	0.0109
Cysteine	0.5	sgol@2−21−0	0.9419	0.0049	0.9419	0.0049	0.8453	0.0078
Glutamic acid	0.1	sgol@2−21−0, msc	0.9751	0.0316	0.9751	0.0316	0.9539	0.0423
Glycine	0.1	sgol@2−21−0, msc	0.9509	0.0049	0.9509	0.0049	0.8988	0.0071
Histidine	0.1	sgol@2−21−0	0.9674	0.0033	0.9673	0.0033	0.9289	0.0049
Isoleucine	0.1	sgol@2−21−0	0.9586	0.0034	0.9586	0.0034	0.9073	0.0051
Leucine	0.1	sgol@2−21−0, msc	0.9835	0.0099	0.9835	0.0099	0.9721	0.0127
Lysine	0.1	sgol@2−21−0, msc	0.9870	0.0073	0.9870	0.0073	0.9808	0.0088
Methionine	0.1	sgol@2−21−0	0.9428	0.0046	0.9428	0.0046	0.8765	0.0066
Phenylalanine	0.1	sgol@2−21−0	0.9810	0.0050	0.9810	0.0050	0.9636	0.0066
Proline	0.1	sgol@2−21−0, msc	0.9659	0.0119	0.9659	0.0119	0.9313	0.0164
Serine	0.1	sgol@2−21−0, msc	0.9804	0.0066	0.9804	0.0066	0.9586	0.0095
Threonine	0.1	sgol@2−21−0	0.9529	0.0064	0.9528	0.0064	0.8943	0.0094
Tyrosine	0.1	sgol@2−21−0	0.9846	0.0051	0.9845	0.0051	0.9717	0.0068
Valine	0.1	sgol@2−21−0, msc	0.9537	0.0069	0.9537	0.0069	0.8949	0.0103

## Data Availability

The figures and tables used to support the findings of this study are included in this article.
